# The Paris Exposition

**Published:** 1899-06

**Authors:** 


### The Paris Exposition.

Paris will soon again be the attraction of the world. Parties are already being formed to visit the Exposition which is to be held there next year, and it will interest not a few of our readers to know that an American boarding house, or as the French prefer to call it a "pension," is to be started for the special benefit of those who wish to reside where straight American is spoken, and where, besides, they will have opportunities of meeting other folks from their own country. It will be conducted by Professor Wisner and his wife, who, while natives of France, have long resided in New York City, and are well acquainted with American ways and customs. They have taken a mansion near the Bois de Boulogne, and they intend fitting it up in such a manner as to insure that their guests will have a comfortable home during their stay in the gay capital. The professor and his wife are well known in medical circles in New York, and having arranged to accommodate a number of prominent doctors and their families, they hope to make their house the American headquarters for the profession. Before leaving for Paris, as he intends doing shortly, Professor Wisner would be pleased to hear from other prospective guests. For the present he may be addressed No. 605 Madison Avenue, New York City.

